# Machine learning–based multicenter prediction of postoperative sepsis in emergency colon cancer: role of surgical approach and inflammatory markers

**DOI:** 10.3389/fonc.2026.1845236

**Published:** 2026-05-13

**Authors:** Wenyi Du, Tao Sun, Wentan Chen, Min Sun, Chengyu Shi, Chao Jiang, Feng Zhan, Yu Zhang

**Affiliations:** 1 Department of Hepatobiliary Surgery, The Affiliated Yixing Hospital of Jiangsu University, Yixing, China; 2 General Surgery Centre, Yixing People’s Hospital Affiliated to Jiangsu University, Yixing, China

**Keywords:** colon cancer, emergency, inflammatory response, machine learning, predictive model, sepsis

## Abstract

**Background:**

Postoperative sepsis occurs at a relatively high incidence following emergency colon cancer surgery, and early identification of high-risk patients is crucial for improving clinical outcomes. However, there is currently a lack of systematic risk prediction models specifically tailored for patients undergoing emergency colon cancer surgery. This study aimed to identify risk factors associated with postoperative sepsis and to develop a clinically applicable machine learning–based prediction model.

**Methods:**

This was a multicenter retrospective cohort study including patients who underwent emergency colon cancer surgery between January 2020 and January 2025. Perioperative variables were systematically collected, encompassing preoperative, intraoperative, and early postoperative data, including demographic characteristics, comorbidities, laboratory findings, and surgical features. Univariate and multivariate logistic regression analyses were first performed to identify independent risk factors. Subsequently, five machine learning algorithms—multilayer perceptron (MLP), random forest (RF), support vector machine (SVM), k-nearest neighbor (KNN), and extreme gradient boosting (XGBoost)—were applied to evaluate feature importance and construct predictive models. Model performance was assessed using receiver operating characteristic (ROC) curves, calibration curves, decision curve analysis (DCA), k-fold cross-validation, and external validation. SHapley Additive exPlanations (SHAP) were used to interpret the contribution of key features.

**Results:**

Surgical approach, intraoperative hypothermia, acidosis, hypoxemia, hypoalbuminemia, and postoperative inflammatory markers (procalcitonin [PCT] and neutrophil-to-lymphocyte ratio [NLR]) were identified as independent high-risk factors for postoperative sepsis in emergency colon cancer patients. All five machine learning models demonstrated good discriminative performance, with the MLP model achieving the best overall performance. It exhibited stable discrimination and low variability in both internal validation and external independent validation cohorts. SHAP analysis further confirmed the contribution of the identified risk factors to model predictions.

**Conclusion:**

The MLP-based risk prediction model developed in this study effectively identifies patients at high risk of postoperative sepsis following emergency colon cancer surgery. It provides a scientific basis for early perioperative intervention and individualized management, and offers valuable support for clinical risk stratification and optimization of therapeutic strategies.

## Introduction

Colon cancer ranks among the most prevalent malignancies of the digestive tract worldwide. According to global cancer statistics, colorectal cancer is the third most common malignancy in terms of incidence and the second leading cause of cancer-related mortality, with over 1.9 million new cases and approximately 900,000 deaths reported globally in 2022. With the acceleration of population aging, the westernization of lifestyles, and shifts in dietary patterns, the disease burden of colorectal cancer continues to rise, exhibiting a particularly rapid upward trend in developing countries. As the predominant subtype of colorectal cancer, colon cancer accounts for approximately 60%–70% of all cases, and its epidemiological characteristics and clinical management strategies carry substantial public health significance (1). From a clinical perspective, the early symptoms of colon cancer are often insidious or atypical—such as altered bowel habits, vague abdominal discomfort, and unexplained anemia—resulting in a substantial proportion of patients failing to receive timely diagnosis at an early stage. A considerable number of patients seek medical attention only upon the onset of acute complications, including intestinal obstruction, perforation, or massive hemorrhage, by which point the disease is frequently at an advanced stage, necessitating urgent surgical intervention (2–4). Compared with elective procedures, patients undergoing emergency colon cancer surgery often present with complex pathophysiological derangements, including severe intra-abdominal infection, tissue ischemia and necrosis, and a pronounced systemic inflammatory response, placing the organism in a heightened state of physiological stress. Moreover, emergency operations are commonly constrained by limited preoperative preparation time, greater technical difficulty, and intraoperative hemodynamic instability, collectively compounding the risk of inadequate tissue perfusion, microcirculatory compromise, and immunosuppression. The confluence of these adverse factors results in a substantially higher incidence of postoperative complications following emergency colon cancer surgery than elective procedures, seriously compromising both short-term prognosis and long-term quality of life (5–7).

Among these adverse outcomes, sepsis represents a particularly formidable threat. Defined as life-threatening organ dysfunction precipitated by a dysregulated host response to infection, sepsis is characterized by systemic inflammation, immune dysregulation, and multi-organ injury (8–10). In patients undergoing emergency colon cancer surgery, the pre-existing inflammatory burden, compounded by intraoperative tissue injury, intensifies the systemic inflammatory response, facilitating pathogen dissemination and heightening susceptibility to sepsis. This condition not only prolongs hospitalization and escalates healthcare resource utilization but also significantly increases postoperative mortality and may induce long-term organ dysfunction, rendering it a pivotal determinant of postoperative prognosis in this patient cohort.

Consequently, the identification of high-risk factors for sepsis is critical for the early recognition of vulnerable patients, optimization of perioperative management, and improvement of clinical outcomes. Conventional risk assessment relies predominantly on clinical acumen, patient history, and laboratory indicators—including inflammatory biomarkers, complete blood counts, and biochemical parameters (11–13). However, such approaches possess inherent limitations: single metrics often fail to encapsulate the multifaceted nature of a patient’s condition, clinical judgment is prone to inter-individual variability, and traditional statistical methods are constrained in addressing high-dimensional, multivariable, and nonlinear interactions, thereby limiting accurate identification of patients at elevated risk for sepsis. In recent years, machine learning techniques have emerged as powerful tools in medical data analysis. Unlike conventional methods, machine learning can effectively process complex, multidimensional clinical datasets, discern nonlinear relationships and variable interactions, and achieve more precise prediction of high-risk patients. Moreover, through feature selection and model interpretability strategies—such as SHapley Additive exPlanations (SHAP)—key predictive factors can be delineated and their relative contributions quantified, offering actionable insights to guide clinical decision-making (14–19).

Guided by these considerations, the present study employed machine learning methodologies to systematically analyze multidimensional data encompassing baseline patient characteristics, intraoperative physiological parameters, surgical approaches, and postoperative inflammatory markers in individuals undergoing emergency colon cancer surgery. The overarching objective was to identify critical risk factors for postoperative sepsis and to construct a clinically applicable predictive model, thereby furnishing a robust scientific foundation for early intervention, individualized perioperative management, and reduction in the incidence of postoperative sepsis and multi-organ dysfunction.

## Materials and methods

### Study design and population

This study was designed as a multicenter retrospective cohort study. Patients who underwent emergency surgery for colon cancer between January 2020 and January 2025 were consecutively enrolled from five hospitals: Wuxi People’s Hospital Affiliated to Nanjing Medical University, Wuxi Second People’s Hospital, Tengzhou Central People’s Hospital, Tengzhou Hospital of Traditional Chinese Medicine, and Gaomi People’s Hospital. Patients from Wuxi People’s Hospital Affiliated to Nanjing Medical University and Wuxi Second People’s Hospital were assigned to the internal dataset for model development and internal validation, whereas patients from Tengzhou Central People’s Hospital, Tengzhou Hospital of Traditional Chinese Medicine, and Gaomi People’s Hospital were included in the external dataset for independent external validation.

### Inclusion and exclusion criteria

Patients were screened in accordance with predefined inclusion and exclusion criteria. The inclusion criteria encompassed: (1) histopathologically confirmed primary colon adenocarcinoma, either via preoperative biopsy or postoperative examination; (2) undergoing emergency surgical intervention prompted by colon cancer–related acute abdominal events—such as intestinal obstruction, perforation, massive gastrointestinal hemorrhage, or intussusception—with procedures performed by surgeons proficient in independently executing the relevant surgical techniques; and (3) age ≥18 years.

Exclusion criteria comprised: (1) fulfillment of Sepsis-3.0 criteria for sepsis or presence of septic shock prior to surgery; (2) concurrent active malignancies or distant metastases of colon cancer; (3) exploratory laparotomy without colon resection; (4) prolonged preoperative use (>3 months) of corticosteroids or other immunosuppressive agents, confirmed HIV infection, or other immunocompromised states; (5) history of major abdominal surgery within 30 days preceding the index procedure; (6) pregnancy or lactation; (7) severe dysfunction of vital organs, including hepatic or renal failure; (8) early postoperative mortality from causes unrelated to sepsis; and (9) complete absence of essential outcome variables.

### Data collection and missing data handling

This retrospective multicenter study utilized clinical data extracted from standardized electronic medical record systems across all participating institutions, encompassing perioperative clinical data of patients undergoing emergency surgery for colon cancer, including preoperative, intraoperative, and postoperative variables. To ensure consistency and reliability, predefined case report forms and uniform variable definitions were applied across centers. Data extraction was conducted by trained investigators and independently verified by at least two researchers to minimize potential errors and inconsistencies.

Preoperative variables included demographic characteristics and medical history: sex, age, body mass index (BMI), American Society of Anesthesiologists (ASA) score, albumin (ALB), Nutritional Risk Screening 2002 (NRS2002) score, family history, drinking history, smoking history, prior surgical history, history of chemotherapy and radiotherapy, anemia, hyperlipidemia, hypertension, diabetes, coronary heart disease (CHD), chronic obstructive pulmonary disease (COPD), serum creatinine (SCr), blood urea nitrogen (BUN), indication for surgery (reason for operation), pathological staging (T stage and N stage), prognostic nutritional index (PNI), tumor location, and tumor size. All preoperative laboratory parameters were obtained within 24 hours prior to surgery.

Intraoperative variables included surgical type, surgical method, operative duration, intraoperative blood loss, oxygen saturation (SpO_2_), blood transfusion, hypothermia, acidosis, and tachycardia.

Postoperative variables primarily consisted of inflammatory and tumor-related biomarkers, including carcinoembryonic antigen (CEA), carbohydrate antigen 19-9 (CA19-9), procalcitonin (PCT), neutrophil-to-lymphocyte ratio (NLR), serum amyloid A (SAA), and C-reactive protein (CRP). All postoperative laboratory measurements were collected within 72 hours after surgery.

Through the comprehensive collection of preoperative, intraoperative, and postoperative variables, this study was able to capture the full spectrum of perioperative clinical characteristics and physiological status, thereby providing robust data support for the identification of high-risk factors for postoperative sepsis and the development of machine learning–based predictive models.

Missing data were systematically assessed prior to analysis and categorized according to the proportion of missingness. Variables with a missing rate <5% were defined as low-missingness variables, those with 5–30% as moderate-to-high missingness variables, and variables with >30% missingness were either excluded or encoded as “missing” indicator variables.

Two complementary imputation strategies were employed based on the degree of missingness. For variables with low missingness, simple imputation was applied, with categorical variables imputed using the mode. For variables with moderate-to-high missingness, multiple imputation was performed. Specifically, for binary variables, logistic regression models were fitted using complete cases to estimate the probability of each outcome, followed by stochastic imputation through random sampling from the predicted probability distribution, thereby accounting for inherent uncertainty.

To prevent data leakage and ensure the validity of model evaluation, all imputation procedures were conducted after dataset partitioning, maintaining strict separation between the training, validation, and external datasets.

### Definition of postoperative sepsis and related clinical variables

Sepsis was defined in accordance with the Third International Consensus Definitions for Sepsis and Septic Shock (Sepsis-3, 2016), which conceptualizes sepsis as life-threatening organ dysfunction arising from a dysregulated host response to infection (20–22). For patients outside the intensive care unit, initial screening employed the quick Sequential Organ Failure Assessment (qSOFA) score, comprising three criteria: respiratory rate ≥22 breaths/min, systolic blood pressure ≤100 mmHg, and altered mental status (Glasgow Coma Scale <15). A qSOFA score ≥2 signaled a high risk of sepsis and prompted further evaluation (23, 24).

Subsequently, a thorough clinical assessment was undertaken. Patients presenting with fever or hypothermia, abnormal leukocyte counts, or elevated inflammatory markers underwent evaluation for suspected or confirmed infection, integrating imaging findings (e.g., intra-abdominal abscess, pneumonia, or surgical site infection) and microbiological evidence from blood, abdominal drainage, sputum, or urine cultures. Organ dysfunction was assessed using the Sequential Organ Failure Assessment (SOFA) score, which quantifies impairment across six organ systems—respiratory, coagulation, hepatic, cardiovascular, neurological, and renal—each scored 0–4, yielding a total of 0–24, with higher scores reflecting more severe dysfunction.

In this study, the observation timeframe for postoperative sepsis was explicitly defined as within 7 days after surgery. During this period, all patients were continuously monitored for vital signs, infection-related indicators, and organ function. In the early postoperative phase, systemic inflammatory response syndrome (SIRS) induced by surgical trauma is common and may present with fever, tachycardia, and leukocytosis, potentially overlapping with the initial manifestations of sepsis. To accurately distinguish between these conditions, we strictly adhered to the Sepsis-3 criteria and implemented dynamic assessment of the SOFA score. Baseline SOFA scores were established preoperatively, and postoperative SOFA scores were reassessed daily or more frequently as clinically indicated. Postoperative sepsis was diagnosed in patients with confirmed or strongly suspected infection who exhibited a ≥2-point increase in SOFA score relative to baseline, provided that the increase was persistent or progressive rather than attributable to a transient fluctuation at a single time point. This dynamic evaluation framework enables differentiation between transient physiological stress responses and sustained, infection-related organ dysfunction. In addition to SOFA score dynamics, longitudinal trends in PCT and CRP, microbiological culture results, and imaging findings were integrated to support diagnostic accuracy. All cases of postoperative sepsis were independently adjudicated by two experienced clinicians based on comprehensive clinical, laboratory, and imaging data, ensuring diagnostic rigor and reliability.

Intraoperative hypothermia was defined as a core temperature <36 °C, continuously monitored via an esophageal probe after endotracheal intubation, with any measurement below this threshold considered hypothermic. Intraoperative hypoxemia was defined as oxygen saturation (SpO_2_) <90% persisting for at least 1 minute, rather than a single, transient drop, with heart rate, blood pressure, and skin perfusion also assessed to comprehensively evaluate overall tissue oxygenation, thereby avoiding the misclassification of fleeting fluctuations as clinically significant hypoxic events. Intraoperative acidosis was defined as arterial blood pH <7.35 confirmed by intraoperative arterial blood gas analysis, requiring that the abnormality persisted throughout surgery or necessitated clinical intervention (e.g., adjustment of ventilatory parameters, administration of sodium bicarbonate), rather than representing an isolated laboratory deviation.

For anatomical classification, the colon was categorized into right- and left-sided segments. The right colon, derived from the midgut, comprised the cecum, ascending colon, and proximal two-thirds of the transverse colon, whereas the left colon, originating from the hindgut, included the distal third of the transverse colon, descending colon, and sigmoid colon. This classification reflects embryological origin and anatomical distinctions, consistent with standard clinical practice in colon surgery.

### Ethical approval and informed consent

This study was conducted in strict accordance with the ethical principles of the Declaration of Helsinki and received independent approval from the Institutional Review Boards (IRBs) of all participating centers, including the Ethics Committee of Wuxi People’s Hospital Affiliated to Nanjing Medical University (approval No. KY22086), Wuxi Second People’s Hospital, Tengzhou Central People’s Hospital, Gaomi People’s Hospital, and Tengzhou Hospital of Traditional Chinese Medicine (approval No. IEC-AF/17-1.1).

All participants were fully informed of the study objectives, scope of data collection, and potential risks and benefits prior to enrollment, and written informed consent was obtained. For patients who could not be re-contacted, a waiver of informed consent was granted by the respective IRBs, on the condition that all data were rigorously anonymized.

All identifiable patient information was removed at the time of data extraction and replaced with coded study identifiers. The dataset was stored on a password-protected secure server with access restricted to authorized research personnel only, in compliance with applicable international standards for data protection and privacy.

### Identification of high-risk factors for postoperative sepsis

A multi-tiered approach was undertaken to elucidate high-risk factors for postoperative sepsis following emergency colon cancer surgery and to construct a robust predictive model. Initially, univariate logistic regression was applied to all candidate variables to identify those significantly associated with the outcome (P < 0.05), with unadjusted odds ratios (ORs) and 95% confidence intervals (CIs) calculated. Variables achieving statistical significance were subsequently incorporated into a multivariate logistic regression model to ascertain independent risk factors (P < 0.05).

To transcend the inherent limitations of conventional logistic regression—such as vulnerability to multicollinearity and restricted capacity to capture nonlinear relationships and complex feature interactions—five machine learning algorithms were employed for feature importance assessment and ranking: eXtreme Gradient Boosting (XGBoost), Random Forest (RF), Support Vector Machine (SVM), K-Nearest Neighbors (KNN), and Multilayer Perceptron (MLP). All models were trained and validated using the internal dataset.

The specifications and feature evaluation methods for each model were as follows:

The XGBoost model, grounded in a gradient-boosted decision tree framework, iteratively fits residuals to optimize the objective function, employing a binary logistic loss function for training. Feature importance was quantified by integrating both the frequency of feature utilization in tree splits and the corresponding gain in information, with “gain” representing the actual contribution of a feature to model performance and serving as the more informative metric.

The Random Forest model leveraged bootstrap resampling to generate multiple decision trees and employed ensemble learning to enhance robustness. Feature importance was assessed via the mean decrease in Gini impurity, which reflects the extent to which a feature reduces node impurity across all trees; higher values denote stronger discriminative capacity.

The SVM model constructs an optimal hyperplane for binary classification, employing a radial basis function (RBF) kernel to address nonlinear relationships. As SVM lacks inherent feature importance metrics, Recursive Feature Elimination (RFE) was applied, iteratively removing the least contributory features based on model weights, thereby producing a ranked hierarchy of predictors.

The KNN model, a nonparametric, instance-based algorithm, classifies samples according to the majority vote of neighboring instances, using Euclidean distance in this study. In the absence of intrinsic feature weighting, permutation importance was employed, whereby the decrement in model performance following random permutation of a single feature indicates its relative importance.

The MLP model, a feedforward neural network, utilized a single hidden layer with a ReLU activation function and a Sigmoid output to yield probability estimates. Training was conducted using the Adam optimizer with early stopping to mitigate overfitting. Feature importance was derived using the connection weight method, integrating input-to-hidden and hidden-to-output weights; larger absolute values denote greater influence on model predictions.

Each model generated a top-10 ranking of influential features. Integrating statistical and machine learning analyses, the final predictive model incorporated clinical variables that satisfied two criteria: (1) identified as independent risk factors in multivariate logistic regression (P < 0.05), and (2) ranked among the top 10 features in at least three of the five machine learning models. This integrative strategy enabled the identification of core predictors underpinning the postoperative sepsis risk model.

### Model development and comparison

Based on the previously identified high-risk factors, five machine learning models were developed, including XGBoost, RF, SVM, KNN, and MLP. All models were trained and optimized using the internal dataset and subsequently evaluated on an external independent dataset to assess generalizability and robustness.

During model training, grid search was employed for systematic hyperparameter tuning to achieve optimal performance. Specifically, an exhaustive search was conducted across predefined hyperparameter spaces, and model performance under different parameter combinations was evaluated. The combination yielding the highest average performance was selected as the final configuration. This approach reduces dependence on a single data split and enhances model stability and generalization capability.

Model discrimination was primarily assessed using ROC curves and the AUC, with an AUC closer to 1 indicating stronger discriminative ability. Stability and generalizability were further evaluated by comparing AUC values across different models on both the internal dataset and the external dataset. Calibration performance was assessed using calibration curves to examine agreement between predicted probabilities and observed outcomes. In addition, the Brier score, defined as the mean squared difference between predicted probabilities and actual outcomes, was calculated to quantify overall prediction error; lower values indicate better calibration.

To evaluate potential clinical utility, DCA was performed to calculate net benefit across a range of threshold probabilities. By comparing the net benefit of each model with the “treat-all” and “treat-none” strategies, the clinical applicability of the models under varying decision-making scenarios was assessed.

To further assess generalization and stability, 10-fold cross-validation was conducted for all five models. The internal dataset was randomly partitioned into 10 subsets, with each subset serving as the validation set in turn while the remaining subsets were used for training. This procedure was repeated across all folds, and the average performance was used to estimate model generalization.

Multiple evaluation metrics were incorporated for comprehensive performance assessment. The Kolmogorov–Smirnov (KS) statistic was used to evaluate the ability to distinguish between positive and negative samples, with higher values indicating better discrimination. Learning curves were plotted to assess model performance across varying training sample sizes, facilitating the identification of potential overfitting or underfitting. Confusion matrices for both training and test sets were also constructed to present distributions of true positives (TP), false positives (FP), true negatives (TN), and false negatives (FN), providing a multidimensional view of classification performance.

Based on this multidimensional evaluation framework, the optimal model was selected by comprehensively considering discrimination, calibration, and clinical utility, and subsequently validated on the external independent dataset. ROC curves, calibration curves, and DCA were constructed to evaluate its discriminative performance, calibration ability, and clinical net benefit in the external population, confirming its generalizability and applicability.

Finally, to enhance interpretability, SHAP analysis was performed on the optimal model. SHAP, derived from Shapley value theory in cooperative game theory, quantifies each feature’s contribution to model predictions. SHAP summary plots were generated to illustrate overall feature importance, impact direction, and distribution across the dataset. SHAP decision plots were also constructed to visualize the cumulative contribution of individual features for single-sample predictions, revealing the model’s decision-making process and key driving factors.

## Results

### Clinical characteristics of patients with colon cancer and identification of risk factors for postoperative sepsis

**Figure 1:**
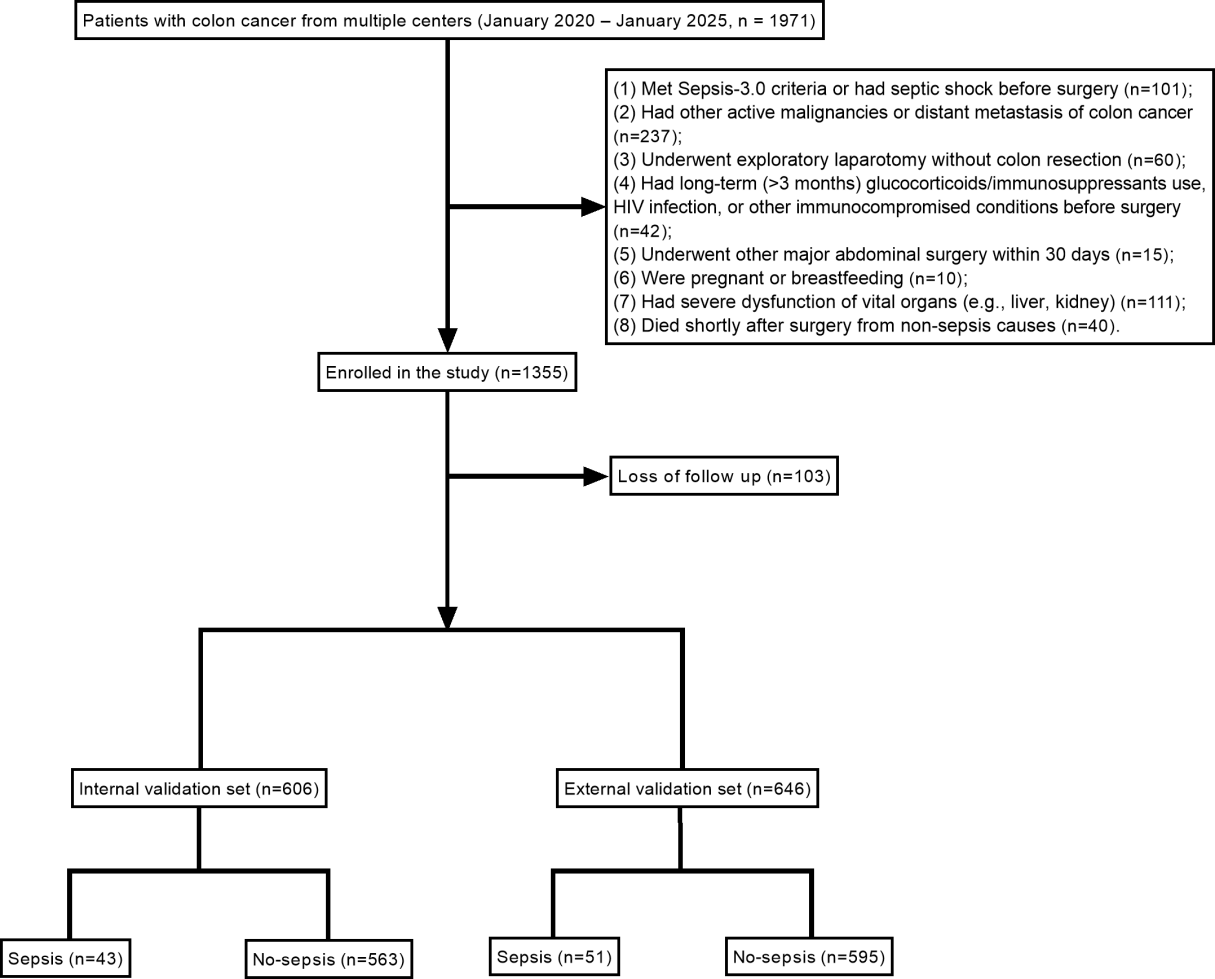
Flow diagram of study selection process according to inclusion and exclusion criteria.

**Figure 2:**
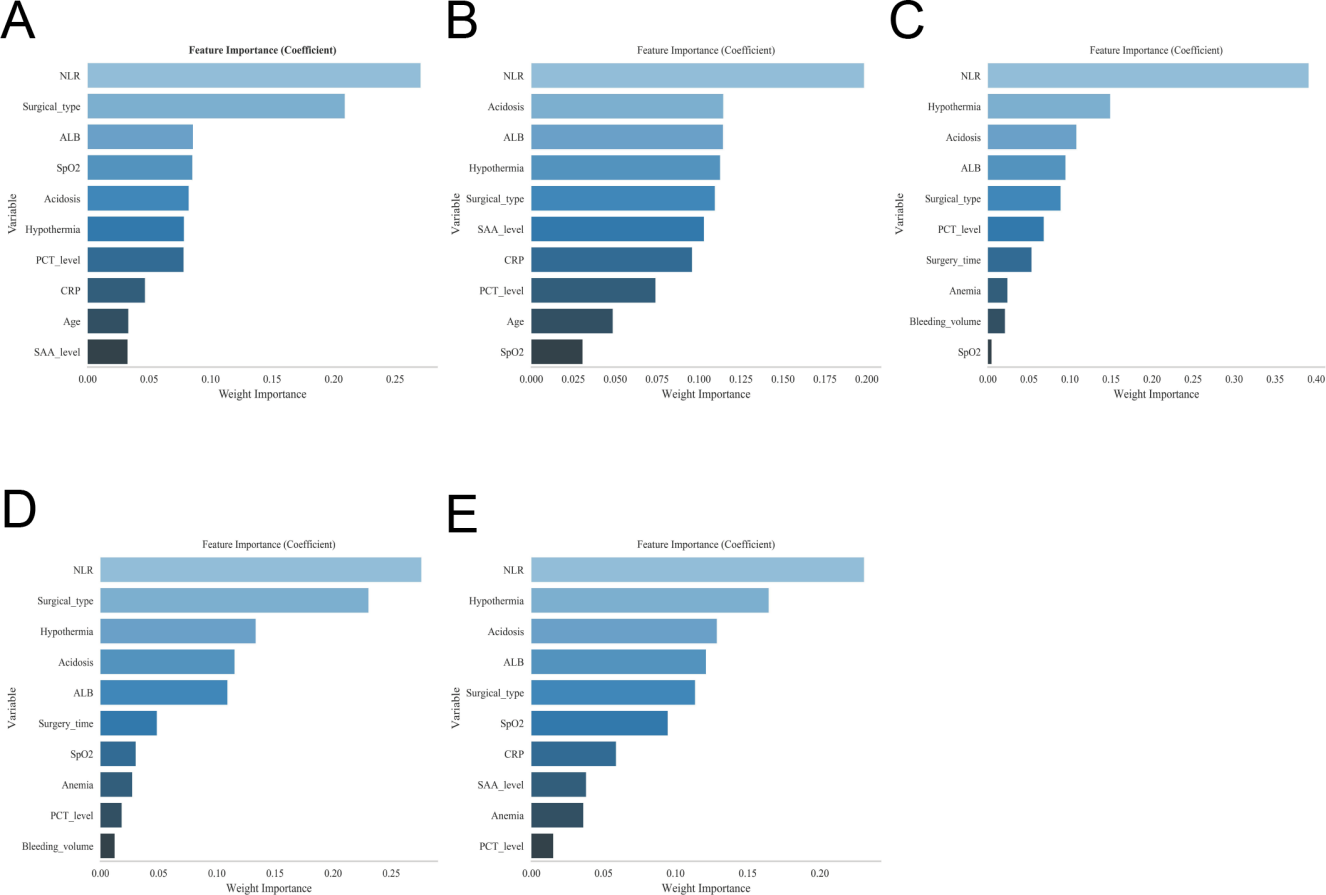
Identification of risk factors for postoperative sepsis using five machine learning models. **(A)** XGBoost, **(B)** random forest, **(C)** support vector machine, **(D)** k-nearest neighbors, and **(E)** MLP.

A total of 1,252 patients with colon cancer were included in this study, comprising 606 patients in the internal dataset and 646 patients in the external validation dataset. In the internal dataset, 43 patients developed postoperative sepsis, corresponding to an incidence of 7.10%. In the external dataset, 51 patients developed sepsis, with an incidence of 7.89% (**Table 1**). Univariate analysis was first performed to identify potential factors associated with postoperative sepsis. Variables significantly associated with the outcome included ALB, anemia, hypertension, CHD, COPD, SCr, BUN, surgical method, operative duration, SpO_2_, hypothermia, acidosis, tumor location, PCT, CRP, SAA, and NLR (all P < 0.05). Subsequently, variables with statistical significance in the univariate analysis were entered into a multivariate logistic regression model. The results demonstrated that ALB, surgical method, intraoperative SpO_2_, hypothermia, acidosis, PCT, NLR, and SAA remained independent predictors of postoperative sepsis (P < 0.05), indicating that these factors may serve as potential independent risk factors (**Figure 1**, **Table 2**). To further refine feature selection, five machine learning models—XGBoost, RF, SVM, MLP, and KNN—were applied. Feature importance rankings across all models consistently identified ALB, surgical method, intraoperative SpO_2_, hypothermia, acidosis, PCT, and NLR among the top ten most important predictors. Notably, these variables were also confirmed as independent predictors in the multivariate regression analysis. Based on their consistent performance across both traditional statistical methods and multiple machine learning models, these factors were selected for inclusion in the final predictive model to further explore high-risk determinants of postoperative sepsis (**Figures 2A–E**). The main characteristics of the dataset used in this study, including the number of samples, number of features, and sample distribution, are presented in **Table 3**. The original dataset utilized in this study is provided in **Supplementary Table 1**.

**Table 1:** Baseline clinical characteristics in the dataset.

Variables		Overall (N = 1252)	Internal validation set (N = 606)	External validation set (N = 646)	P-value
Sex	Female	536 (42.812)	218 (35.974)	318 (49.226)	<0.001
	Male	716 (57.188)	388 (64.026)	328 (50.774)	
Age	<60	438 (34.984)	178 (29.373)	260 (40.248)	<0.001
	≥60	814 (65.016)	428 (70.627)	386 (59.752)	
BMI	<25 kg/m^2^	895 (71.486)	427 (70.462)	468 (72.446)	0.437
	≥25 kg/m^2^	357 (28.514)	179 (29.538)	178 (27.554)	
ASA	<3	906 (72.364)	428 (70.627)	478 (73.994)	0.183
	≥3	346 (27.636)	178 (29.373)	168 (26.006)	
ALB	≥30g/L	951 (75.958)	465 (76.733)	486 (75.232)	0.535
	<30g/L	301 (24.042)	141 (23.267)	160 (24.768)	
NRS2002 score	<3	929 (74.201)	410 (67.657)	519 (80.341)	<0.001
	≥3	323 (25.799)	196 (32.343)	127 (19.659)	
Family history	No	1047 (83.626)	511 (84.323)	536 (82.972)	0.518
	Yes	205 (16.374)	95 (15.677)	110 (17.028)	
Drinking history	No	969 (77.396)	446 (73.597)	523 (80.960)	0.002
	Yes	283 (22.604)	160 (26.403)	123 (19.040)	
Smoking history	No	1022 (81.629)	482 (79.538)	540 (83.591)	0.064
	Yes	230 (18.371)	124 (20.462)	106 (16.409)	
Surgical history	No	1017 (81.230)	469 (77.393)	548 (84.830)	<0.001
	Yes	235 (18.770)	137 (22.607)	98 (15.170)	
Chemotherapy	No	1092 (87.220)	532 (87.789)	560 (86.687)	0.56
	Yes	160 (12.780)	74 (12.211)	86 (13.313)	
Radiotherapy	No	1110 (88.658)	538 (88.779)	572 (88.545)	0.896
	Yes	142 (11.342)	68 (11.221)	74 (11.455)	
Anemia	No	1142 (91.214)	546 (90.099)	596 (92.260)	0.177
	Yes	110 (8.786)	60 (9.901)	50 (7.740)	
Hyperlipidemia	No	1029 (82.188)	495 (81.683)	534 (82.663)	0.651
	Yes	223 (17.812)	111 (18.317)	112 (17.337)	
Hypertension	No	976 (77.955)	466 (76.898)	510 (78.947)	0.382
	Yes	276 (22.045)	140 (23.102)	136 (21.053)	
Diabetes	No	1120 (89.457)	532 (87.789)	588 (91.022)	0.063
	Yes	132 (10.543)	74 (12.211)	58 (8.978)	
CHD	No	1148 (91.693)	553 (91.254)	595 (92.105)	0.586
	Yes	104 (8.307)	53 (8.746)	51 (7.895)	
COPD	No	1131 (90.335)	525 (86.634)	606 (93.808)	<0.001
	Yes	121 (9.665)	81 (13.366)	40 (6.192)	
SCr	<1.2 mg/dL	1098 (87.700)	514 (84.818)	584 (90.402)	0.003
	≥1.2 mg/dL	154 (12.300)	92 (15.182)	62 (9.598)	
BUN	<20 mg/dL	1106 (88.339)	523 (86.304)	583 (90.248)	0.03
	≥20 mg/dL	146 (11.661)	83 (13.696)	63 (9.752)	
T stage	T1~T2	864 (69.010)	414 (68.317)	450 (69.659)	0.608
	T3~T4	388 (30.990)	192 (31.683)	196 (30.341)	
N stage	N0	893 (71.326)	425 (70.132)	468 (72.446)	0.366
	N1~N2	359 (28.674)	181 (29.868)	178 (27.554)	
PNI	No	1069 (85.383)	494 (81.518)	575 (89.009)	<0.001
	Yes	183 (14.617)	112 (18.482)	71 (10.991)	
Tumor location	Left-sided colon	704 (56.230)	329 (54.290)	375 (58.050)	0.18
	Right-sided colon	548 (43.770)	277 (45.710)	271 (41.950)	
Tumor size	<5 cm	999 (79.792)	486 (80.198)	513 (79.412)	0.729
	≥5 cm	253 (20.208)	120 (19.802)	133 (20.588)	
Reason for operation	Intestinal obstruction	1030 (82.268)	504 (83.168)	526 (81.424)	0.935
	Intestinal perforation	75 (5.990)	33 (5.446)	42 (6.502)	
	Hemorrhage	68 (5.431)	32 (5.281)	36 (5.573)	
	Intussusception	36 (2.875)	17 (2.805)	19 (2.941)	
	Others	43 (3.435)	20 (3.300)	23 (3.560)	
Surgical method	Primary anastomosis	664 (53.035)	304 (50.165)	360 (55.728)	0.057
	Primary anastomosis with stoma	190 (15.176)	96 (15.842)	94 (14.551)	
	Simple stoma	119 (9.505)	53 (8.746)	66 (10.217)	
	Hartmann’s procedure	279 (22.284)	153 (25.248)	126 (19.505)	
Surgical type	Open surgery	980 (78.275)	491 (81.023)	489 (75.697)	0.022
	Laparoscopic surgery	272 (21.725)	115 (18.977)	157 (24.303)	
Surgery time	<270 min	932 (74.441)	431 (71.122)	501 (77.554)	0.009
	≥270 min	320 (25.559)	175 (28.878)	145 (22.446)	
Intraoperative bleeding	<100 ml	947 (75.639)	443 (73.102)	504 (78.019)	0.043
	≥100 ml	305 (24.361)	163 (26.898)	142 (21.981)	
Intraopera hypothermia	No	1076 (85.942)	517 (85.314)	559 (86.533)	0.535
	Yes	176 (14.058)	89 (14.686)	87 (13.467)	
Intraopera acidosis	No	1092 (87.220)	535 (88.284)	557 (86.223)	0.275
	Yes	160 (12.780)	71 (11.716)	89 (13.777)	
Intraoperative tachycardia	No	974 (77.796)	441 (72.772)	533 (82.508)	<0.001
	Yes	278 (22.204)	165 (27.228)	113 (17.492)	
Intraoperative SpO_2_	≥90%	1136 (90.735)	568 (93.729)	568 (87.926)	<0.001
	<90%	116 (9.265)	38 (6.271)	78 (12.074)	
Blood transfusion	No	1138 (90.895)	548 (90.429)	590 (91.331)	0.579
	Yes	114 (9.105)	58 (9.571)	56 (8.669)	
PCT level	<0.05 ng/ml	989 (78.994)	501 (82.673)	488 (75.542)	0.002
	≥0.05 ng/ml	263 (21.006)	105 (17.327)	158 (24.458)	
CRP level	<10 mg/l	1023 (81.709)	513 (84.653)	510 (78.947)	0.009
	≥10 mg/l	229 (18.291)	93 (15.347)	136 (21.053)	
SAA level	<10 mg/l	1029 (82.188)	517 (85.314)	512 (79.257)	0.005
	≥10 mg/l	223 (17.812)	89 (14.686)	134 (20.743)	
NLR	<3	1034 (82.588)	513 (84.653)	521 (80.650)	0.062
	≥3	218 (17.412)	93 (15.347)	125 (19.350)	
CEA level	<5 ng/ml	1024 (81.789)	486 (80.198)	538 (83.282)	0.158
	≥5 ng/ml	228 (18.211)	120 (19.802)	108 (16.718)	
CA199 level	<37 U/mL	1057 (84.425)	512 (84.488)	545 (84.365)	0.952
	≥37 U/mL	195 (15.575)	94 (15.512)	101 (15.635)	
Sepsis	No	1158 (92.492)	563 (92.904)	595 (92.105)	0.592
	Yes	94 (7.508)	43 (7.096)	51 (7.895)	

OR, odds ratio; CI, confidence interval; BMI, body mass index; ASA, The American Society of Anesthesiologists; ALB, albumin; PCT, procalcitonin; CRP, C-reactive protein; SAA, serum amyloid A; NRS2002, nutrition risk screening 2002; COPD, chronic obstructive pulmonary disease; CHD, coronary heart disease; SCr, serum creatinine; BUN, blood urea nitrogen; PNI, perineural invasion; CEA, carcinoembryonic antigen; CA199, carbohydrate antigen 19-9; SPO_2_, percutaneous arterial oxygen saturation.

**Table 2:** Univariate and multivariate logistic regression analysis for postoperative sepsis.

Variables		Univariate analysis		Multivariate analysis	
		OR, 95% CI	P-value	OR, 95% CI	P-value
Sex	Female	Reference			
	Male	0.944 [0.497,1.794]	0.861		
Age	<60	Reference			
	≥60	0.551 [0.293,1.038]	0.065		
BMI	<25 kg/m^2^	Reference			
	≥25 kg/m^2^	0.918 [0.460,1.831]	0.808		
ASA	<3	Reference			
	≥3	0.712 [0.343,1.479]	0.363		
ALB	≥30g/L	Reference		Reference	
	<30g/L	9.397 [4.746,18.607]	0	11.195 [2.434,71.524]	0.004
NRS2002 score	<3	Reference			
	≥3	0.702 [0.346,1.425]	0.328		
Family history	No	Reference			
	Yes	1.468 [0.680,3.170]	0.328		
Drinking history	No	Reference			
	Yes	1.379 [0.709,2.681]	0.344		
Smoking history	No	Reference			
	Yes	0.881 [0.398,1.950]	0.754		
Surgical history	No	Reference			
	Yes	0.9 [0.420,1.925]	0.785		
Chemotherapy	No	Reference			
	Yes	1.439 [0.616,3.364]	0.4		
Radiotherapy	No	Reference			
	Yes	1.916 [0.850,4.322]	0.117		
Anemia	No	Reference		Reference	
	Yes	3.109 [1.447,6.680]	0.004	4.037 [0.539,33.354]	0.174
Hyperlipidemia	No	Reference			
	Yes	1.021 [0.460,2.266]	0.96		
Hypertension	No	Reference		Reference	
	Yes	2.339 [1.229,4.450]	0.01	6.232 [0.961,49.729]	0.061
Diabetes	No	Reference			
	Yes	1.18 [0.480,2.901]	0.718		
CHD	No	Reference		Reference	
	Yes	4.928 [2.355,10.312]	0	2.518 [0.332,18.047]	0.354
COPD	No	Reference		Reference	
	Yes	2.421 [1.167,5.020]	0.017	1.361 [0.126,10.906]	0.781
SCr	<1.2 mg/dL	Reference		Reference	
	≥1.2 mg/dL	3.002 [1.519,5.932]	0.002	1.342 [0.225,7.168]	0.733
BUN	<20 mg/dL	Reference		Reference	
	≥20 mg/dL	3.052 [1.520,6.129]	0.002	5.606 [0.971,39.058]	0.061
T stage	T1~T2	Reference			
	T3~T4	1.785 [0.952,3.344]	0.071		
N stage	N0	Reference			
	N1~N2	0.902 [0.453,1.800]	0.771		
PNI	No	Reference			
	Yes	1.182 [0.550,2.541]	0.668		
Tumor location	Left-sided colon	Reference		Reference	
	Right-sided colon	1.901 [1.009,3.582]	0.047	2.222 [0.443,13.099]	0.343
Tumor size	<5 cm	Reference			
	≥5 cm	1.631 [0.811,3.280]	0.17		
Reason for operation	Intestinal obstruction	Reference			
	Intestinal perforation	2.179 [0.719,6.604]	0.168		
	Hemorrhage	2.257 [0.743,6.854]	0.151		
	Intussusception	3.386 [0.922,12.428]	0.066		
	Others	1.756 [0.389,7.921]	0.464		
Surgical method	Primary anastomosis	Reference			
	Primary anastomosis with stoma	1.225 [0.524,2.863]	0.639		
	Simple stoma	1.404 [0.505,3.901]	0.515		
	Hartmann’s procedure	0.842 [0.376,1.886]	0.676		
Surgical type	Open surgery	Reference		Reference	
	Laparoscopic surgery	9.109 [4.713,17.603]	0	9.110 [1.658,68.431]	0.016
Surgery time	<270 min	Reference		Reference	
	≥270 min	5.916 [3.042,11.506]	0	2.637 [0.566,12.578]	0.209
Intraoperative bleeding	<100 ml	Reference			
	≥100 ml	1.867 [0.985,3.541]	0.056		
Intraopera hypothermia	No	Reference		Reference	
	Yes	10.829 [5.600,20.939]	0	18.332 [3.323,153.947]	0.002
Intraopera acidosis	No	Reference		Reference	
	Yes	15.61 [7.934,30.713]	0	16.741 [3.546,111.317]	0.001
Intraoperative tachycardia	No	Reference			
	Yes	1.833 [0.967,3.475]	0.063		
Intraoperative SpO_2_	≥90%	Reference		Reference	
	<90%	4.874 [2.138,11.115]	0	13.946 [1.046,181.351]	0.039
Blood transfusion	No	Reference			
	Yes	1.594 [0.642,3.953]	0.315		
PCT level	<0.05 ng/ml	Reference		Reference	
	≥0.05 ng/ml	15.015 [7.496,30.078]	0	5.107 [1.054,30.171]	0.05
CRP level	<10 mg/l	Reference		Reference	
	≥10 mg/l	20.875 [10.194,42.745]	0	3.565 [0.761,19.724]	0.116
SAA level	<10 mg/l	Reference		Reference	
	≥10 mg/l	19.713 [9.746,39.875]	0	6.522 [1.368,40.921]	0.026
NLR	<3	Reference		Reference	
	≥3	45.654 [19.422,107.315]	0	9.173 [1.941,57.068]	0.009
CEA level	<5 ng/ml	Reference			
	≥5 ng/ml	1.432 [0.700,2.930]	0.326		
CA199 level	<37 U/mL	Reference			
	≥37 U/mL	1.988 [0.964,4.099]	0.063		

OR, odds ratio; CI, confidence interval; BMI, body mass index; ASA, The American Society of Anesthesiologists; ALB, albumin; PCT, procalcitonin; CRP, C-reactive protein; SAA, serum amyloid A; NRS2002, nutrition risk screening 2002; COPD, chronic obstructive pulmonary disease; CHD, coronary heart disease; SCr, serum creatinine; BUN, blood urea nitrogen; PNI, perineural invasion; CEA, carcinoembryonic antigen; CA199, carbohydrate antigen 19-9; SPO_2_, percutaneous arterial oxygen saturation.

**Table 3:** Main characteristics of the dataset.

Characteristic	Internal dataset	External dataset	Total
Number of samples	606	646	1252
Number of features	41	41	41
Positive cases (sepsis)	43	51	94
Negative cases (non-sepsis)	563	595	1158
Proportion of positive cases (%)	7.1	7.89	7.51

### Model development and comparison

**Figure 3:**
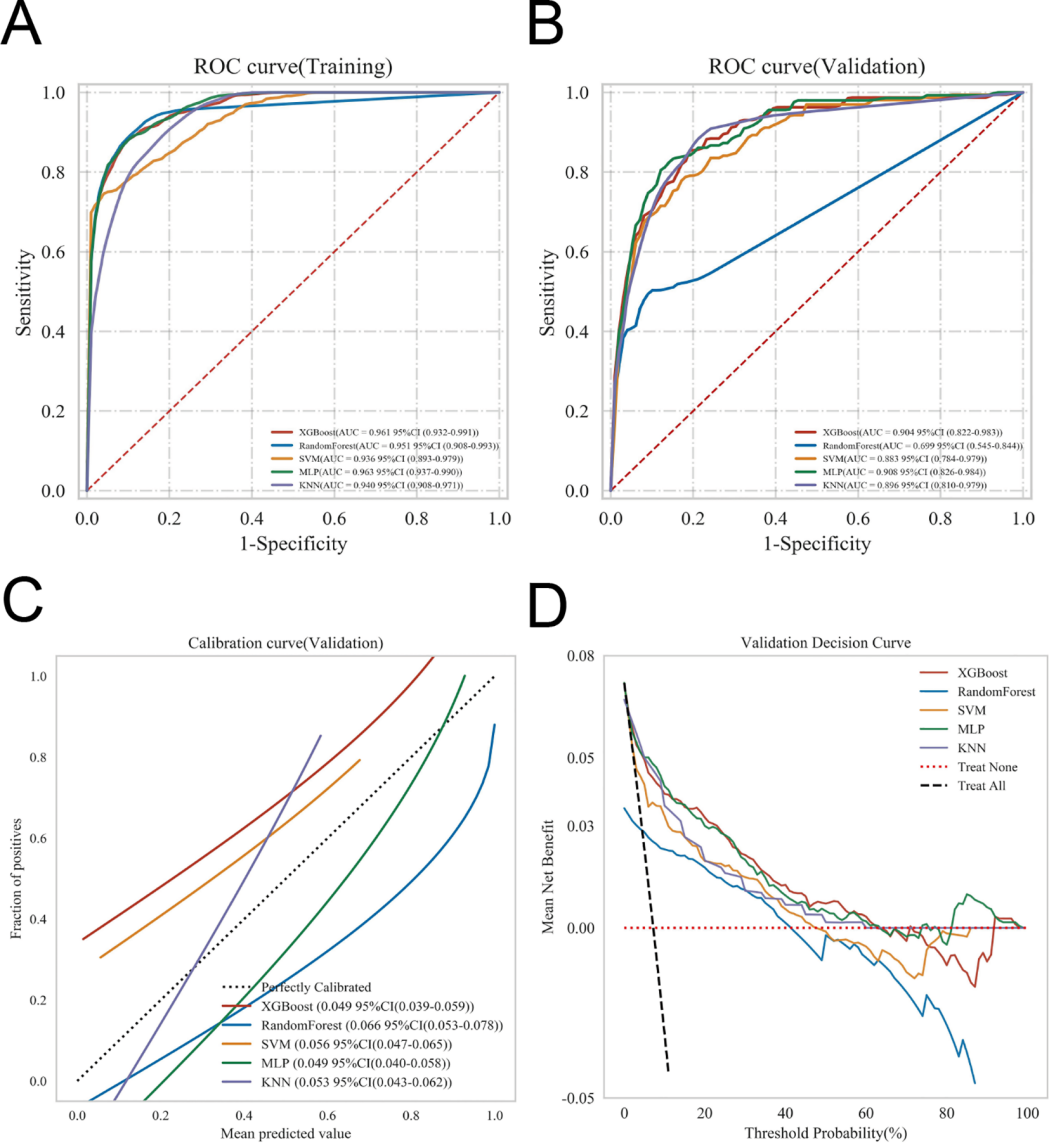
Comparative evaluation of five machine learning models in terms of accuracy, stability, and clinical utility. The predictive performance and clinical applicability of the five machine learning models were comprehensively assessed. **(A)** Receiver operating characteristic (ROC) curves in the training cohort, illustrating the discriminative ability of each model. **(B)** ROC curves in the validation cohort, demonstrating the generalizability of the models. **(C)** Calibration curves, evaluating the agreement between predicted probabilities and observed outcomes. **(D)** Decision curve analysis (DCA), quantifying the net clinical benefit across a range of threshold probabilities to assess the clinical utility of each model.

ROC curve analysis demonstrated that the MLP model achieved the best discriminative performance among the five evaluated machine learning models. In the training set, the MLP model yielded an AUC of 0.963 (95% CI: 0.937–0.990), while in the internal validation set, the AUC was 0.908 (95% CI: 0.826–0.984) (**Table 4**; **Figures 3A, B**). Calibration curves indicated good agreement between predicted probabilities and observed outcomes across all models (**Figure 3C**). DCA further demonstrated that all models provided net clinical benefit across a range of threshold probabilities, supporting potential clinical utility (**Figure 3D**).

**Table 4:** Comparison of selected features across five machine learning models.

Machine models		AUC (95% CI)	Accuracy (95% CI)	Sensitivity (95% CI)	Specificity (95% CI)	F1 Score (95% CI)	Kappa (95% CI)
KNN	training set	0.940 (0.908-0.971)	0.815 (0.783-0.847)	0.914 (0.868-0.960)	0.808 (0.771-0.845)	0.419 (0.371-0.466)	0.349 (0.298-0.400)
	validation set	0.896 (0.810-0.979)	0.81 (0.787-0.834)	0.892 (0.850-0.934)	0.804 (0.777-0.830)	0.401 (0.364-0.438)	0.327 (0.291-0.362)
XGBoost	training set	0.961 (0.932-0.991)	0.897 (0.882-0.912)	0.901 (0.884-0.917)	0.897 (0.880-0.914)	0.557 (0.518-0.595)	0.509 (0.466-0.552)
	validation set	0.904 (0.822-0.983)	0.876 (0.852-0.900)	0.735 (0.639-0.832)	0.888 (0.867-0.910)	0.462 (0.378-0.545)	0.404 (0.313-0.496)
RF	training set	0.951 (0.908-0.993)	0.902 (0.880-0.924)	0.895 (0.866-0.924)	0.903 (0.879-0.927)	0.573 (0.522-0.624)	0.527 (0.470-0.585)
	validation set	0.699 (0.545-0.844)	0.845 (0.813-0.877)	0.515 (0.396-0.635)	0.873 (0.838-0.908)	0.324 (0.256-0.393)	0.253 (0.174-0.332)
SVM	training set	0.936 (0.893-0.979)	0.91 (0.860-0.960)	0.818 (0.767-0.869)	0.917 (0.861-0.974)	0.618 (0.518-0.718)	0.579 (0.465-0.694)
	validation set	0.883 (0.784-0.979)	0.853 (0.805-0.902)	0.695 (0.604-0.786)	0.865 (0.807-0.924)	0.419 (0.368-0.471)	0.356 (0.298-0.415)
MLP	training set	0.963 (0.937-0.990)	0.91 (0.897-0.922)	0.878 (0.853-0.903)	0.912 (0.898-0.927)	0.582 (0.545-0.618)	0.539 (0.498-0.579)
	validation set	0.908 (0.826-0.984)	0.884 (0.866-0.901)	0.732 (0.658-0.805)	0.896 (0.876-0.916)	0.471 (0.407-0.535)	0.416 (0.348-0.483)

CI, confidence interval; KNN, k-nearest neighbor; XGBoost, extreme gradient boosting; RF, random forest; SVM, support vector machine; MLP, multilayer perceptron; AUC, area under the curve.

**Figure 4:**
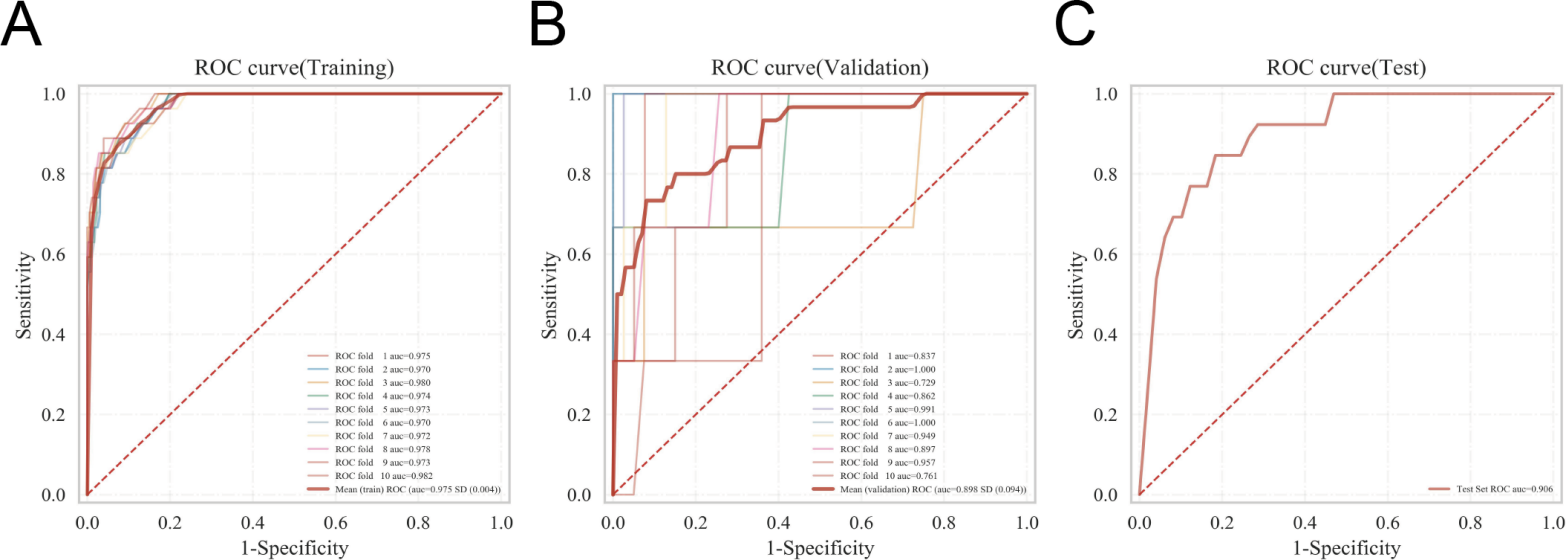
K-fold cross-validation of the multilayer perceptron (MLP) model. The robustness and generalizability of the MLP model were evaluated using k-fold cross-validation. **(A)** Receiver operating characteristic (ROC) curves in the training set across all folds, demonstrating the model’s internal consistency. **(B)** ROC curves in the validation set, illustrating the model’s stability during hyperparameter tuning. **(C)** ROC curves in the test set, reflecting the model’s generalizability on unseen data.

To evaluate model generalizability, 10-fold cross-validation was performed using the internal dataset. For the MLP model, the mean cross-validation AUC was 0.8985 ± 0.0938, and the test set AUC was 0.9065, with an overall accuracy of 0.8242 (**Figures 4A–C**). Comparative performance metrics for other models were as follows: RF, cross-validation AUC 0.8789 ± 0.1190, test AUC 0.9001, accuracy 0.8736; SVM, cross-validation AUC 0.8958 ± 0.1060, test AUC 0.8596, accuracy 0.8956; KNN, cross-validation AUC 0.7772 ± 0.1810, test AUC 0.8232, accuracy 0.8921; and XGBoost, cross-validation AUC 0.8782 ± 0.1356, test AUC 0.8979, with the highest accuracy of 0.9286. Overall, the MLP model exhibited lower variability and consistent AUC across cross-validation folds, indicating robust performance.

**Figure 5:**
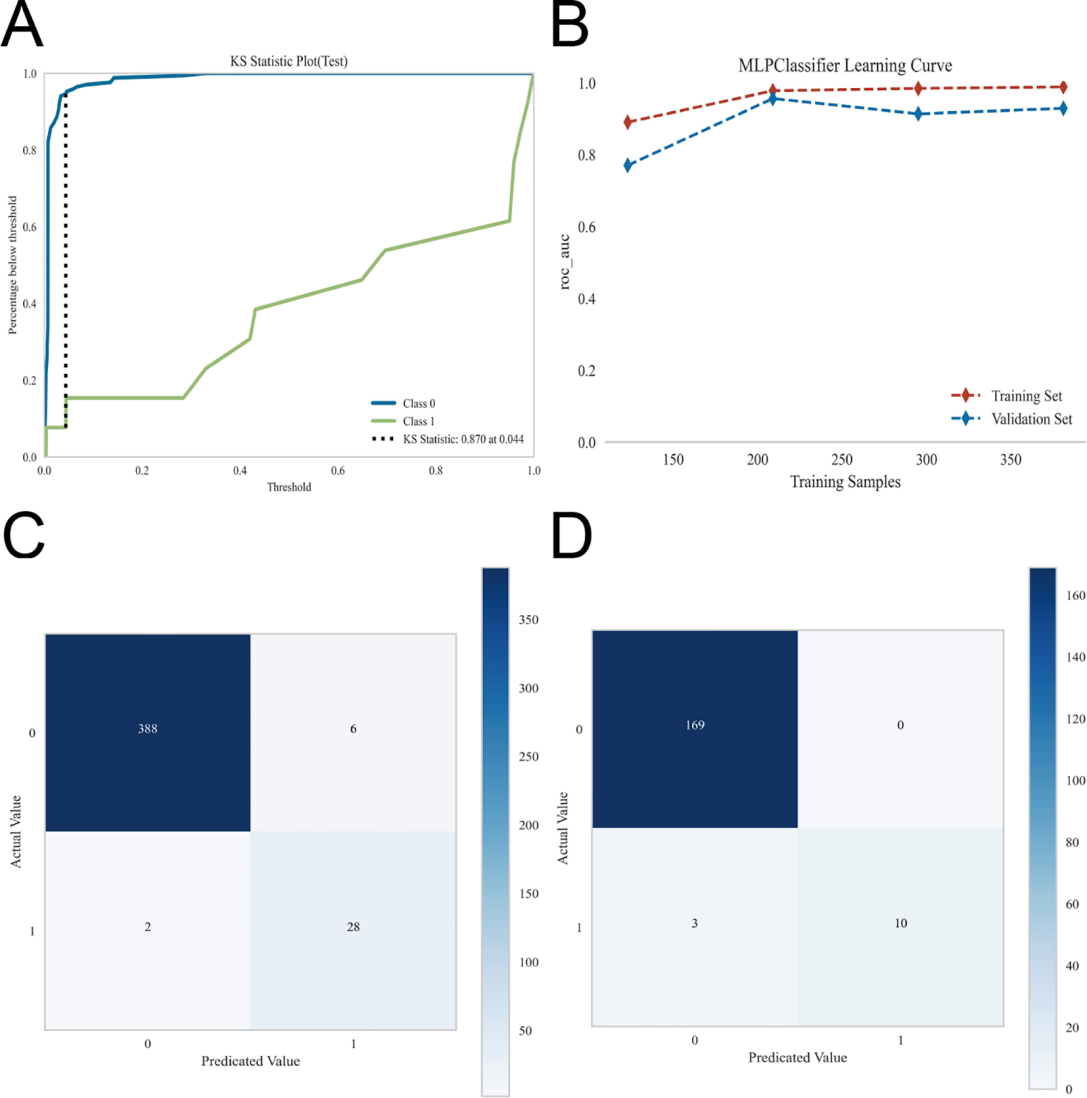
Multidimensional evaluation of the multilayer perceptron (MLP) model. A comprehensive assessment of the MLP model was performed from multiple perspectives to evaluate its discriminative ability, learning behavior, and classification performance. **(A)** Kolmogorov–Smirnov (KS) curve, illustrating the maximum separation between the cumulative distribution functions of positive and negative cases, reflecting the model’s discriminative power. **(B)** Learning curve, depicting the evolution of training and validation performance over training iterations, demonstrating the model’s fitting status and the presence of overfitting or underfitting. **(C)** Confusion matrix in the training set, presenting the detailed classification outcomes including true positives, true negatives, false positives, and false negatives. **(D)** Confusion matrix in the test set.

KS analysis of the MLP model yielded a KS statistic of 0.870 at an optimal cutoff threshold of 0.044, reflecting strong separation between positive and negative cases. Learning curve analysis showed convergence of training and validation performance with increasing sample size, suggesting minimal overfitting and adequate model fit. Confusion matrix analysis further confirmed high classification accuracy and balanced sensitivity and specificity across both training and test sets, indicating reliable predictive performance (**Figures 5A–D**).

**Figure 6:**
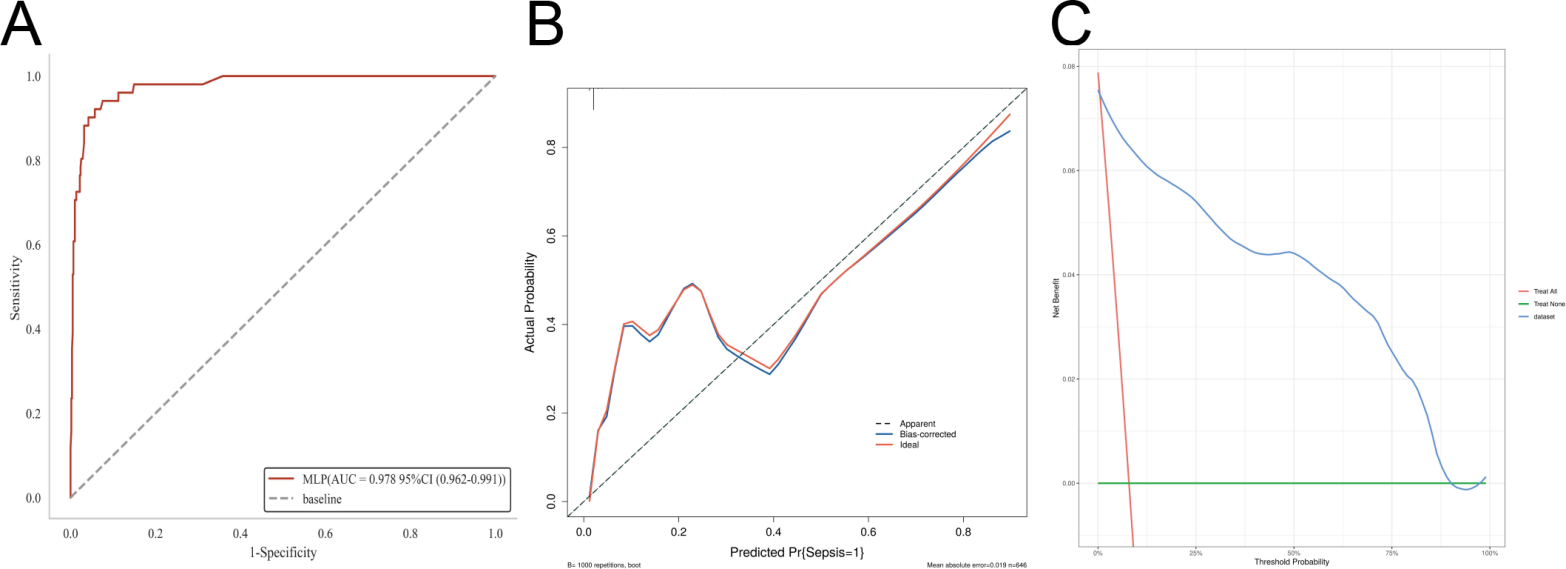
External validation of the multilayer perceptron (MLP) model. The generalizability and clinical applicability of the MLP model were evaluated using an independent external dataset. **(A)** Receiver operating characteristic (ROC) curve, demonstrating the discriminative performance of the model in the external validation cohort, with the area under the curve (AUC) quantifying the overall predictive accuracy. **(B)** Calibration curve, assessing the agreement between predicted probabilities and observed outcomes, with the calibration slope and intercept reflecting the model’s reliability. **(C)** Decision curve analysis (DCA), quantifying the net clinical benefit across a range of threshold probabilities to evaluate the clinical utility of the model in the external setting.

External validation of the MLP model reinforced its robustness and generalizability. ROC analysis demonstrated excellent discriminative ability, with an AUC of 0.978 (95% CI: 0.962–0.991). Calibration curves and DCA indicated good agreement between predicted and observed outcomes and potential clinical utility in the independent cohort (**Figures 6A–C**). Collectively, these results confirm that the MLP model provides stable, accurate, and clinically applicable prediction of postoperative sepsis in patients undergoing emergency colon cancer surgery.

### Model interpretability via SHAP analysis

**Figure 7:**
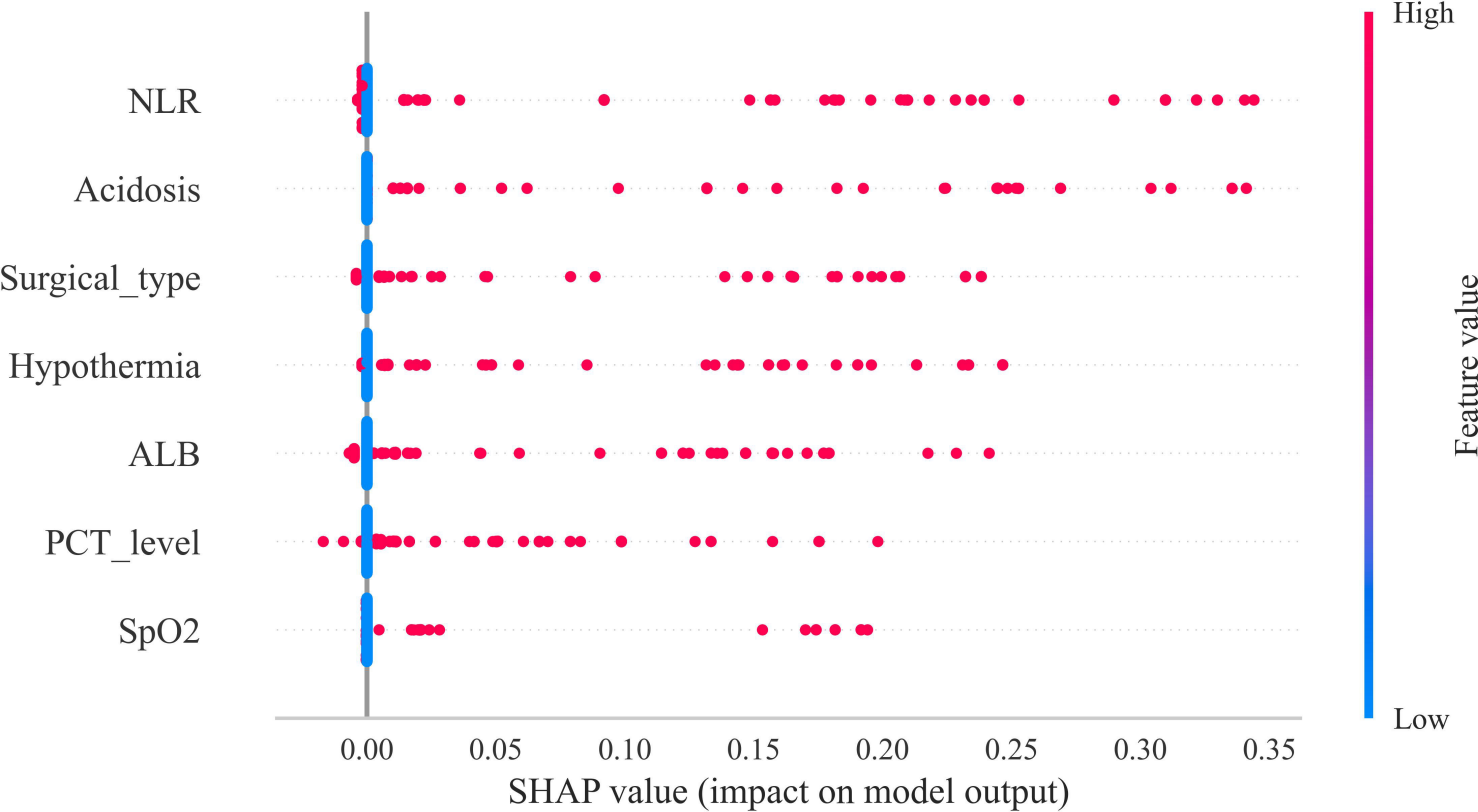
Global interpretability of the multilayer perceptron (MLP) model using SHAP analysis. To enhance the interpretability of the MLP model, Shapley additive explanations (SHAP) analysis was employed. The SHAP summary plot provides a global overview of feature importance, illustrating the overall contribution of each feature to the model’s predictions. The color gradient represents the feature value (high vs. low), while the position on the x-axis indicates the direction and magnitude of the impact—positive or negative—on the predicted outcome. This visualization enables a comprehensive understanding of the key drivers influencing postoperative sepsis risk at the population level.

To elucidate the interpretability of the predictive model, SHAP analysis was performed. The SHAP summary plot identified the relative importance of postoperative sepsis predictors in descending order: elevated postoperative NLR, intraoperative acidosis, surgical type, intraoperative hypothermia, hypoalbuminemia, elevated postoperative PCT, and intraoperative hypoxemia. These results indicate that both perioperative inflammatory and metabolic dysregulation markers, as well as surgery-related factors, play critical roles in the development of postoperative sepsis. Specifically, NLR and PCT elevations reflect systemic inflammatory responses, while intraoperative acidosis, hypothermia, and hypoxemia indicate perioperative physiological stress. These factors may serve as potential targets for early postoperative monitoring and intervention (**Figure 7**).

**Figure 8:**
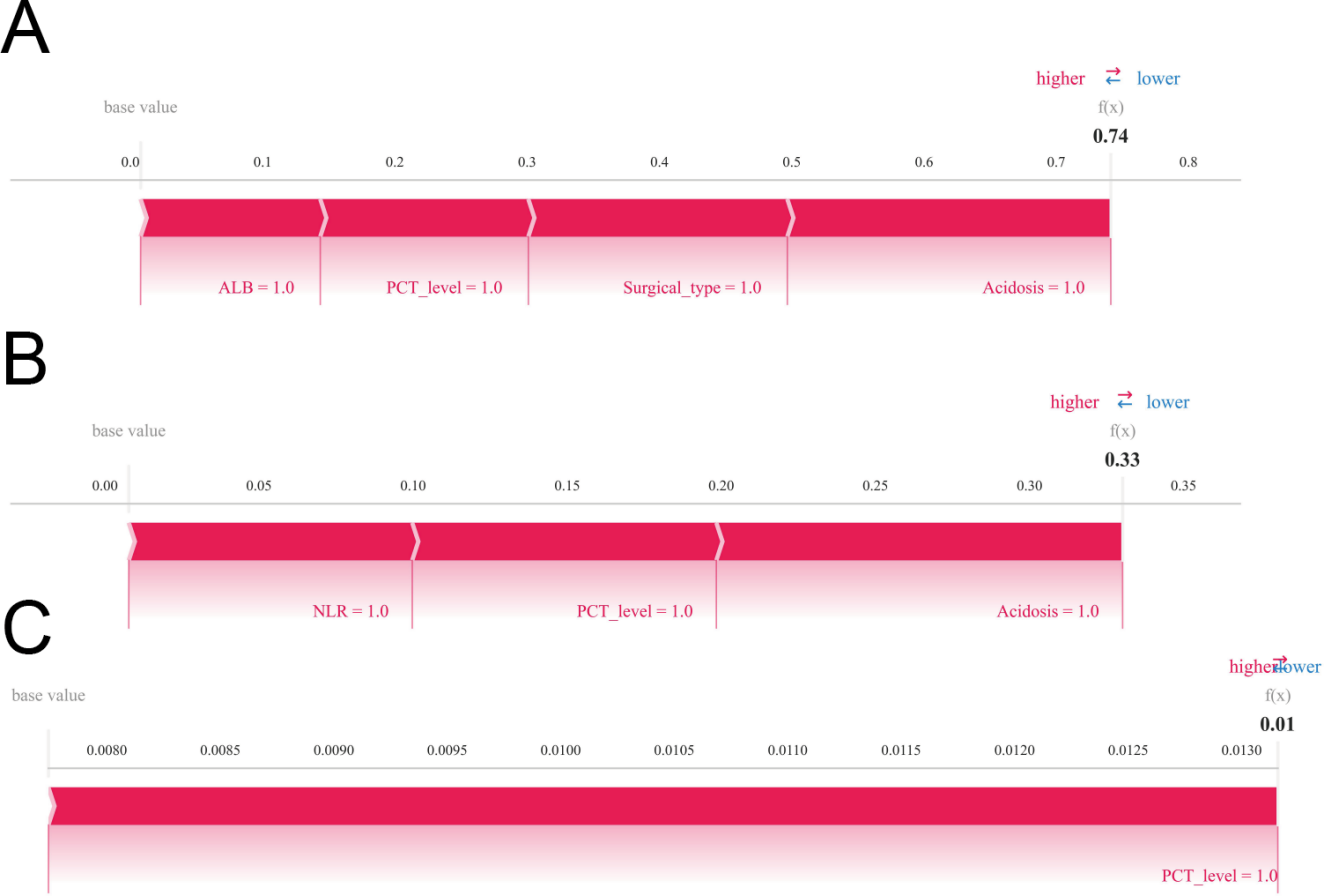
Individual-level interpretability of the MLP model using SHAP force plots. **(A–C)** Representative examples from three individual patients. To enable personalized risk assessment, SHAP force plots were generated to illustrate the interpretability of the MLP model at the individual level. **(A–C)** Representative cases from three distinct patients. Each force plot visualizes the baseline prediction (expected value) and demonstrates how individual features contribute to pushing the final prediction toward a higher or lower risk of postoperative sepsis. Features contributing to increased risk are shown in red, while those reducing risk are shown in blue. These plots facilitate intuitive understanding of the model’s decision-making process for individual clinical scenarios.

To further explore individualized predictive capability, SHAP decision plots were constructed for three representative patients (**Figures 8A–C**).

Patient 1 was classified as high-risk, with a predicted probability of 0.74. The SHAP force plot highlighted hypoalbuminemia, elevated postoperative PCT, complex or highly invasive surgical procedures, and intraoperative acidosis as the main contributors to the high-risk prediction. These features reflect a perioperative state characterized by enhanced inflammation, metabolic imbalance, and substantial surgical trauma, collectively elevating the likelihood of postoperative sepsis. This example demonstrates that early identification of high-risk patients by the model may guide targeted interventions addressing hypoalbuminemia, inflammation, and acid-base disturbances, potentially improving clinical outcomes.

Patient 2 had a predicted probability of 0.33, indicating moderate risk. The SHAP force plot revealed that perioperative inflammatory markers (NLR, PCT) and intraoperative acidosis were the primary drivers of the predicted risk, whereas hypoalbuminemia and surgical type contributed minimally. This patient’s perioperative state was relatively stable, and the model successfully identified moderate risk factors, highlighting its potential utility in personalized monitoring and tailored interventions.

Patient 3 was predicted as low-risk, with a probability of 0.01. The SHAP plot showed that only mildly elevated postoperative PCT slightly increased risk, while other factors such as acidosis, hypoalbuminemia, or surgical trauma had negligible contribution. This indicates a stable perioperative physiological state, emphasizing the model’s ability to conservatively identify low-risk individuals and avoid unnecessary interventions.

## Discussion

In this study, we employed five widely utilized machine learning algorithms—MLP, RF, SVM, KNN, and XGBoost—to model and predict high-risk factors for postoperative sepsis in patients undergoing emergency colon cancer surgery. These models were deliberately selected to encompass diverse algorithmic paradigms, thereby enabling a comprehensive exploration of complex inter-variable relationships while ensuring model robustness. Specifically, MLP, as a feedforward artificial neural network, captures intricate nonlinear interactions among features via nonlinear activation functions, making it particularly suited for high-dimensional categorical variables and multifactorial clinical datasets (25–27). RF and XGBoost, as ensemble tree-based methods, construct numerous decision trees with either voting or gradient optimization, demonstrating resilience to noisy data and high-dimensional features while providing feature importance metrics (28, 29). SVM, through the construction of an optimal hyperplane and kernel-induced mapping to higher-dimensional spaces, excels in small-sample, high-dimensional, and nonlinearly separable classification tasks. KNN, a simple instance-based lazy learning algorithm, is intuitive but susceptible to noise and distance metric limitations in high-dimensional or imbalanced datasets. In our analyses, MLP exhibited superior discriminative performance among all models, with predicted probabilities closely aligning with observed outcomes and demonstrating substantial clinical net benefit in decision curve analysis. During internal cross-validation, the MLP model showed minimal fluctuation and stable generalizability. By contrast, RF and XGBoost, though accurate overall, displayed slightly greater variability; SVM exhibited limited capacity to model complex nonlinear interactions; and KNN proved relatively unstable in high-dimensional, imbalanced contexts. Considering discriminative power, calibration, and generalizability collectively, MLP emerged as the most stable and clinically promising model and was thus selected as the final predictive framework.

Leveraging feature selection and integrative analysis from the final MLP model, this study identified surgical modality, intraoperative hypothermia, acidosis, hypoxemia, hypoalbuminemia, and postoperative inflammatory markers (PCT, NLR) as core high-risk factors for postoperative sepsis in emergency colon cancer patients. These variables not only demonstrated statistically significant associations but also held high importance scores within the machine learning model, underscoring their critical role in clinical risk stratification. From a pathophysiological and perioperative management perspective, elucidating the mechanisms by which these factors contribute to sepsis may inform early identification and targeted interventions.

Hypoalbuminemia represents a pivotal predisposing factor for postoperative sepsis in this patient population, mediated through several mechanisms. Primarily, hypoalbuminemia reflects malnutrition and inadequate protein reserves, compromising immune defense—including impaired phagocytic function, reduced complement activity, and diminished antimicrobial capacity—thereby lowering resistance to intra- and postoperative infections. Furthermore, decreased serum albumin diminishes plasma colloid osmotic pressure, precipitating a cascade of events. As the principal protein maintaining intravascular oncotic pressure, albumin depletion facilitates fluid extravasation into interstitial spaces, resulting in tissue edema (30–32). In emergency colon cancer patients, this manifests as peritoneal and intestinal wall edema, thickening the bowel wall and narrowing luminal diameter. This restricts the operative field, complicating laparoscopic or open surgical maneuvers, while simultaneously expanding interstitial pathways that enable bacterial and inflammatory mediator dissemination, promoting systemic infection. Edema also impairs local microcirculation, reducing tissue oxygenation and immune cell infiltration, thereby compromising local infection clearance (33–35). Critically, hypoalbuminemic patients exhibit diminished stress resilience, predisposing them to immune dysregulation and organ dysfunction under the high-trauma, high-infection-risk context of emergency surgery. Collectively, hypoalbuminemia constitutes both a preoperative risk marker and a mechanistic contributor to postoperative sepsis, warranting proactive nutritional optimization, plasma protein correction, and vigilant infection surveillance to mitigate sepsis and associated complications.

Although prior studies (36, 37) have highlighted the advantages of minimally invasive surgery, particularly laparoscopy, in elective colon procedures—such as reduced trauma, less postoperative pain, faster gastrointestinal recovery, and shorter hospitalization—our findings reveal a significant association between laparoscopic approach and postoperative sepsis in emergency colon cancer surgery. This may reflect the unique pathophysiological and operative challenges in this cohort. Laparoscopy necessitates CO_2_ pneumoperitoneum to maintain visualization, yet emergency patients frequently present with intestinal perforation, obstruction, or peritonitis, often accompanied by intra-abdominal bacterial contamination or exudates (38). Pneumoperitoneum may facilitate bacterial and inflammatory mediator dissemination while perturbing local microcirculation and immune responses, exacerbating systemic inflammation and infection risk (39, 40). Additionally, bowel distension, edema, and contamination constrain the operative field and obscure anatomical landmarks, requiring extensive tissue manipulation and repeated instrument handling, which prolongs operative time, augments tissue trauma, and heightens bacterial spread, collectively elevating postoperative infection risk (41–43). Finally, laparoscopic techniques may be limited in performing thorough peritoneal lavage or clearing infectious foci compared with open surgery, particularly in deep or concealed regions, leaving residual contaminants that perpetuate inflammatory stimuli and promote sepsis (44, 45).

Beyond baseline patient factors and surgical modality, intraoperative physiological perturbations critically influence postoperative sepsis. Our analyses identified intraoperative acidosis and hypoxemia as salient risk factors, often co-occurring and reflecting profound tissue hypoperfusion and metabolic derangement. Emergency colon cancer patients frequently present with bowel obstruction, ischemic necrosis, or peritonitis, leading to tissue hypoxia and lactic acid accumulation, thereby inducing metabolic acidosis (46–48). Concurrent hypoxemia exacerbates cellular oxygen deprivation, shifting metabolism toward anaerobic pathways, amplifying lactate production and acidosis in a vicious cycle (49, 50). Both acidosis and hypoxemia impair immune function, with reduced phagocytic and bactericidal capacities of neutrophils and macrophages, suppressed lymphocyte activity, and diminished clearance of pathogens and inflammatory mediators, facilitating infection spread and systemic inflammatory response (51–53). These conditions also compromise cardiovascular performance and microcirculatory perfusion, impairing oxygen delivery, diminishing organ function, and exacerbating systemic inflammation (54, 55). Mitochondrial energy metabolism is further disrupted, reducing ATP production, tissue repair capacity, and resilience to injury. In emergency colon surgery, the coexistence of intra-abdominal infection or severe inflammation with acidosis and hypoxemia accelerates progression from local infection to systemic inflammatory response and sepsis (56, 57). Therefore, vigilant intraoperative monitoring and correction of acid-base status and oxygenation are imperative to mitigate postoperative sepsis and multiorgan dysfunction.

Intraoperative hypothermia, prevalent in emergency colon procedures, arises from anesthesia-induced thermoregulatory impairment, peritoneal exposure, infusion of unwarmed fluids, and prolonged operative duration. Our findings link intraoperative hypothermia with postoperative sepsis, suggesting its role in immune suppression, coagulopathy, and microcirculatory dysfunction. Hypothermia diminishes neutrophil chemotaxis, phagocytosis, and bactericidal activity, impairs macrophage and T-cell responses, and weakens local infection clearance, facilitating systemic inflammatory escalation (58–60). It also impairs hemostasis through reduced platelet function and thrombin activity, prolonging bleeding and heightening transfusion requirements, further exacerbating immunosuppression and infection susceptibility (61–63). Additionally, hypothermia compromises tissue repair and wound healing by suppressing cellular metabolism, collagen synthesis, and fibroblast proliferation, undermining the immune barrier at surgical sites. In the context of emergency colon surgery, where patients often present with obstruction, peritonitis, or contamination, intraoperative hypothermia may precipitate immune imbalance, accelerating local infections into systemic sepsis.

Postoperative inflammatory response constitutes a fundamental substrate for sepsis development. We evaluated multiple inflammatory markers, including CRP, NLR, PCT, and SAA, employing machine learning and logistic regression analyses. PCT and NLR emerged as the most critical predictive factors. PCT, a specific biomarker for bacterial infection, rises early and proportionally to infection severity and inflammatory burden, offering high sensitivity and specificity for early sepsis detection in postoperative emergency patients (64, 65). NLR reflects the balance between inflammatory activity and immune competence; elevated neutrophils and decreased lymphocytes denote a “pro-inflammatory, immunosuppressed” state, predisposing to rapid infection propagation and systemic inflammatory response (66, 67). Compared with CRP or SAA, PCT and NLR exhibit faster kinetics, dynamic responsiveness, and stronger correlation with infection severity, enhancing early identification of high-risk patients. Mechanistically, elevated PCT signals systemic inflammatory dysregulation, whereas high NLR indicates compromised immune control, jointly constituting the physiological substrate for sepsis development (68–70). Dynamic monitoring of PCT and NLR can guide timely antimicrobial intervention, immunomodulatory support, and perioperative management optimization, thereby mitigating postoperative sepsis and organ dysfunction.

The predictive model developed in this study is not, in essence, a purely preoperative risk stratification tool, but rather a perioperative risk identification and early warning instrument. This clinical positioning must be clearly articulated to avoid confusion with traditional preoperative risk assessment models. Preoperative risk stratification models typically rely on static assessment based on patients’ baseline characteristics at admission (e.g., age, comorbidities, nutritional status), and their role lies in assisting preoperative decision-making and patient counseling. However, such models cannot capture the impact of surgical trauma and early postoperative physiological changes on complication risk. The development of postoperative sepsis is a dynamically evolving pathophysiological process, influenced not only by preoperative baseline susceptibility but also, and more critically, by the degree of intraoperative stress and the trajectory of early postoperative inflammatory responses. Therefore, relying solely on preoperative information for risk discrimination entails inherent limitations. The core value of our model lies in integrating preoperative baseline characteristics, intraoperative events, and early postoperative variables (particularly the dynamic changes of inflammatory biomarkers within 72 hours after surgery), thereby achieving a transition from “static stratification” to “dynamic identification.” In the early postoperative period, the patient’s physiological response to surgical stress has begun to manifest, and inflammatory indicators have started to change, while sepsis may not yet have fully developed or worsened—this window period represents precisely the optimal opportunity for clinical intervention. By performing dynamic risk assessment during this phase, our model can assist clinicians in identifying high-risk patients before sepsis progresses, thereby securing a valuable time window for timely intervention. Based on the early warning signals provided by the model, clinicians can implement a series of targeted interventions: upgrading the level of nursing care to enhance bedside monitoring, increasing the frequency of vital sign monitoring to detect early signs of deterioration, initiating early infection surveillance (e.g., imaging studies and microbiological cultures) to identify the source of infection, and administering proactive supportive therapy (e.g., fluid resuscitation, adjustment of anti-infective treatment, and organ function support). The core of this strategy lies in driving a paradigm shift in clinical management—from passive treatment after sepsis onset to proactive prevention based on risk early warning, thereby interrupting the occurrence and progression of postoperative sepsis.

Several limitations of this study should be acknowledged.

First, a notable class imbalance existed in the dataset, with relatively few positive cases (sepsis, n = 94) compared to negative cases (non-sepsis, n = 1158). Such imbalance may bias model training toward the majority class and potentially affect performance evaluation. To mitigate this issue, we employed a comprehensive evaluation framework beyond accuracy, including the area under the AUC, KS statistic, calibration curves, Brier score, and DCA, which together provide a more robust assessment of discrimination, calibration, and clinical utility under imbalanced conditions. In addition, 10-fold cross-validation was performed to ensure that minority class samples were utilized across multiple training iterations, thereby reducing sampling bias and improving model stability and generalizability. Nevertheless, class imbalance cannot be completely eliminated, and future studies will explore advanced strategies such as SMOTE, Tomek links, and cost-sensitive learning to further enhance model performance.

Second, this study was retrospective in design, which inherently introduces potential selection and information biases. Although we incorporated an independent external validation cohort from multiple centers to improve generalizability, the performance observed in retrospective datasets may not fully reflect real-world clinical applicability. In particular, factors such as real-time data availability, workflow integration, and clinician–model interaction may influence the effectiveness of the model in practice. Furthermore, this study lacks prospective validation in a real-time clinical setting. Future research should focus on multicenter prospective cohort studies to evaluate model performance after deployment and to assess its impact on clinical decision-making and patient outcomes.

Third, residual confounding cannot be entirely excluded. Despite incorporating a wide range of perioperative variables and applying multivariate analysis and machine learning approaches, unmeasured or unrecorded factors—such as surgeon experience, intraoperative decision-making, and variability in postoperative management—may have influenced the results. Future studies should expand the scope of variable collection and consider causal inference methods, such as propensity score techniques or instrumental variable analysis, to further strengthen confounding control.

Fourth, intraoperative oxygenation assessment based on pulse oximetry may be subject to measurement limitations, particularly under conditions of hypoperfusion. Factors such as massive blood loss, vasoconstriction induced by vasoactive agents, and hypothermia may compromise signal accuracy, potentially leading to underestimation, signal dropout, or delayed detection of hypoxemia. Although efforts were made to mitigate this limitation—such as periodic adjustment of probe placement, cross-validation with arterial blood gas analysis, and integration of hemodynamic parameters and clinical judgment—measurement bias cannot be completely excluded.

Despite the strong predictive performance of the proposed model, the potential risk of demographic bias warrants careful consideration. Machine learning models in healthcare may exhibit unequal performance across subgroups defined by sex, age, or other demographic characteristics, which may lead to disparities in risk stratification and clinical decision-making. Previous studies have demonstrated that such biases may exist in colorectal cancer–related prediction models and other oncology applications, potentially affecting the fairness and generalizability of model outputs (71, 72). More broadly, concerns regarding algorithmic fairness in healthcare have been widely discussed, emphasizing the need to balance predictive accuracy with equitable performance across populations (73, 74). In the present study, although sex and age were included as candidate variables during initial feature selection, they were not retained as independent predictors in the final model. This may partially mitigate their direct influence; however, it does not preclude the possibility that demographic information is indirectly encoded through correlated clinical variables. Recent advances in machine learning have proposed various strategies to address algorithmic bias, including fairness-constrained optimization, reweighting techniques, and federated learning frameworks aimed at reducing subgroup disparities (75–77). Future studies should incorporate subgroup analyses stratified by key demographic variables and adopt fairness metrics—such as equal opportunity and demographic parity—to systematically evaluate model equity. The integration of bias mitigation strategies may further enhance the ethical robustness and clinical applicability of predictive models in diverse patient populations.

## Conclusion

In conclusion, this multicenter study systematically evaluated the influence of perioperative clinical variables on postoperative sepsis in emergency colon cancer patients. By integrating logistic regression with five machine learning algorithms, we established an MLP-centered risk prediction model. Surgical modality, intraoperative hypothermia, acidosis, hypoxemia, hypoalbuminemia, and postoperative inflammatory markers (PCT, NLR) were identified as pivotal high-risk factors. The MLP model demonstrated superior discriminative ability, calibration, and generalizability, maintaining robust performance in independent external validation, underscoring its potential utility for early clinical identification of high-risk patients.

## Data Availability

The original contributions presented in the study are included in the article/**Supplementary Material**. Further inquiries can be directed to the corresponding authors.

## Supplementary material

Supplementary Table 1 Raw data.
